# Infection-Associated Cancers

**DOI:** 10.1155/2020/4979131

**Published:** 2020-03-16

**Authors:** Hironori Yoshiyama, Keiji Ueda, Jun Komano, Hisashi Iizasa

**Affiliations:** ^1^Department of Microbiology, Shimane University, Izumo, Shimane, Japan; ^2^Division of Virology, Department of Microbiology and Immunology, Graduate School of Medicine, Osaka University, Osaka, Japan; ^3^Department of Microbiology, Osaka University of Pharmaceutical Sciences, Takatsuki, Japan

After the finding by Peyton Rous that filtered extracts from chicken sarcoma generated new sarcoma, numbers of pathogens have been found to be oncogenic. Microorganisms and their metabolites, as well as chronic inflammation, have also been considered to cause cancers. Almost 20% of all cancers worldwide are estimated to be associated with infections. However, we believe cancers caused by infection include many effective preventive measures, which include a potential target for novel cancer diagnostics, therapeutic approaches, and the possibility of prevention by vaccination. In this special issue, we intend to correct papers concerning current knowledge of infection-associated cancers, spanning basic biology, and potential clinical applications. The focus will be on molecular mechanisms to understand infection-attributable cancers, on tumor microenvironment including tumor immune response, on development of novel biomarkers for diagnosis and for predicting prognosis, and on animal models for studying infection-associated cancers. The purpose of this special issue is to present the recent progress in these exciting fields. A brief summary of all accepted papers is provided below.

M. A. Hernández-Luna et al. reviewed the suggested bacterial molecular mechanisms and their possible role in development and progression of gastrointestinal neoplasms, focusing mainly on colon neoplasms, where the bacteria *Fusobacterium nucleatum*, *Escherichia coli*, *Bacteroides fragilis*, and *Salmonella enterica* infect.

The paper by G. Zhang et al. has developed an ELISA assay to detect infection of human beta retrovirus (HBRV). As a result, anti-HBRV antibodies were detected with a significant difference in patients with breast cancer and primary biliary cholangitis compared to controls.

F. I. Bussière et al. showed a novel way for *H. pylori* to promote genome instabilities through the inhibition of TERT levels and telomerase activity in a mice model. The inflammation and ROS-mediated mechanism could play an important role in the early steps of gastric carcinogenesis.

The review by C. K. Chan et al. focused on current situation on human papillomavirus epidemiology in developing countries, where incidence of cervical cancer is high.

The paper by Y. S. Li et al. focused on the multiple functions of deubiquitinating enzymes (DUBs) in RIG-I-like receptors and stimulators of interferon gene-mediated antiviral signaling pathways, oncovirus regulation of NF-*κ*B activation, oncoviral life cycle, and the potential of DUB inhibitors as therapeutic strategies.

A. R. Adams et al. investigated the size of HPV prevalence in female sex workers (FSWs) to provide information for future assessment of the impact of vaccine introduction in Ghana. They found a high HPV prevalence with high risk-HPV genotypes (HPV-16, HPV-35, HPV-33/39/-68, HPV-52/51/59, and HPV-18) among FSWs in the Greater Accra Region and concluded the efficacy of preventable vaccines.

L. S. D. Libera et al. investigated HPV prevalence, genotype distribution, and prognosis aspect in anal cancers in the Midwestern Region of Brazil by a retrospective study. They also reported that gender, histological type, and the presence of distant metastasis were observed as prognostic factors.

F. D. Felice et al. reviewed the evidence-based literature supporting the deintensification strategies in HPV-related oropharyngeal squamous cell carcinoma management, including radiotherapy dose and/or volume reduction, replacement of cisplatin radiosensitising chemotherapy, and the use of transoral surgery. They aimed at raising clinicians in describing the clinical data, the therapeutic implication, and the most promising treatment strategies in HPV-related oropharyngeal cancer scenarios.

